# Supplementary material for: Quality Specific Associations of Carbohydrate Consumption and Frailty Index
Source: Nutrients. 2022 Nov 29;14(23):5072. doi: 10.3390/nu14235072 (PMC9736578; doi:10.3390/nu14235072)
Supplement: Supplementary file 1 [file nutrients-14-05072-s001.zip › nutrients-2039012-supplementary.pdf]

Table S1. Characteristic difference in those with and without follow up data

	No follow up		Longitudinal Analysis		p-value
n	208		816		
Frailty Index	0.15	(0.12)	0.1	(0.06)	<0.001
Age (years)	74.84	(9.65)	73.78	(7.33)	0.084
Sex (%men)	104	(50.00)	397	(48.70)	0.788
Diabetes	34	(16.30)	100	(12.30)	0.148
Fasting glucose (mg/dL)	92.89	(22.15)	91.81	(17.19)	0.448
HBA1C (%)	5.94	(0.74)	5.84	(0.56)	0.07
BMI (kg/m2)	27.14	(5.12)	26.87	(4.45)	0.452
Total energy (kcal)	2008.95	(728.43)	1939.5	(657.46)	0.184
Glycemic load (g/day)	113.05	(47.05)	106.14	(41.53)	0.037
Total grain (serv/day)	6.18	(2.96)	5.97	(2.57)	0.307
Whole grains (serv/day)	1.77	(1.28)	1.82	(1.10)	0.513
Nonwhole grain (serv/day)	4.41	(2.42)	4.15	(2.11)	0.112
Fiber:Carbohydrate	0.09	(0.03)	0.1	(0.02)	0.62
Fiber (g/day)	21.86	(8.74)	20.68	(7.75)	0.055
% energy from Carbohydrate	47.56	(8.48)	45.95	(6.96)	0.004
% energy from PUFA	7.73	(2.19)	7.96	(1.59)	0.078
% energy from MUFA	12.38	(2.73)	12.81	(2.02)	0.01
% energy from SFA	10.63	(2.87)	11.08	(2.20)	0.014

Table S2. Interactions between carbohydrate intake and time on trajectories of frailty index.

	Men & Women			Men			Women		
	b	(SE)	p	b	(SE)	p	b	(SE)	p
% Carbohydrate									
Med*follow-up time	0.0001	(0.001)	0.938	-0.0009	(0.001)	0.402	0.0011	(0.001)	0.324
High*follow-up time	0.0003	(0.001)	0.710	-0.0009	(0.001)	0.442	0.0015	(0.001)	0.204
Glycemic Load									
Med*follow-up time	0.0004	(0.001)	0.570	-0.0010	(0.001)	0.400	0.0013	(0.001)	0.178
High*follow-up time	0.0001	(0.001)	0.879	-0.0017	(0.001)	0.136	0.0021	(0.001)	0.067
Total Grains									
Med*follow-up time	-0.0001	(0.001)	0.867	-0.0002	(0.001)	0.863	0.0000	(0.001)	0.993
High*follow-up time	-0.0009	(0.001)	0.259	-0.0011	(0.001)	0.328	-0.0005	(0.001)	0.675
Whole Grains									
Med*follow-up time	-0.0004	(0.001)	0.597	-0.0006	(0.001)	0.586	-0.0002	(0.001)	0.857
High*follow-up time	-0.0012	(0.001)	0.114	-0.0011	(0.001)	0.310	-0.0012	(0.001)	0.249
Non-whole grains									
Med*follow-up time	0.0003	(0.001)	0.640	0.0009	(0.001)	0.445	0.0000	(0.001)	0.980
High*follow-up time	0.0001	(0.001)	0.907	0.0001	(0.001)	0.950	0.0007	(0.001)	0.566
Fiber:Carbohydrate									
Med*follow-up time	-0.0009	(0.001)	0.235	-0.0002	(0.001)	0.857	-0.0020	(0.001)	0.098
High*follow-up time	-0.0015	(0.001)	0.051	-0.0004	(0.001)	0.738	-0.0030	(0.001)	0.009

Data represents beta estimates from carbohydrate*time term in the mixed effects model
